# Cerium-Doped ZnO Thin Films for Photocatalysts

**DOI:** 10.3390/ma19091739

**Published:** 2026-04-24

**Authors:** Pavlina Bancheva-Koleva, Stephan Kozhukharov, Christian Girginov, Ivo Banchev, Plamen Petkov, Tamara Petkova, Georgi Avdeev

**Affiliations:** 1 Department of Physics, University of Chemical Technology and Metallurgy, 8 Kl. Ohridski Blvd., 1756 Sofia, Bulgaria; s.kozhukharov@uctm.edu (S.K.);; 2 Institute of Electrochemistry and Energy Systems, Bulgarian Academy of Sciences, Block 11, Acad. G. Bonchev Str., 1113 Sofia, Bulgaria; 3 Department of Physical Chemistry, University of Chemical Technology and Metallurgy, 1797 Sofia, Bulgaria; girginov@uctm.edu; 4 Institute of Physical Chemistry, Bulgarian Academy of Sciences, Block 11, Acad. G. Bonchev Str., 1113 Sofia, Bulgaria; g_avdeev@ipc.bas.bg

**Keywords:** cerium doped, ZnO, thin films, photocatalysts

## Abstract

In this work, Ce-doped ZnO thin films at various contents of cerium were deposited on glass substrates by thermal vacuum evaporation to study the influence of Ce concentration on their optical, structural, morphological, and photocatalytic behavior. Pure ZnO and Ce-doped ZnO films doped with 2% and 5% Ce were characterized by SEM, XRD, AFM, UV–VIS spectroscopy, and ellipsometry. The XRD analysis confirmed that all the films retained the hexagonal wurtzite structure, while Ce incorporation induced lattice strain and reduced crystallite size, particularly at higher doping levels. SEM and AFM studies showed that films with 2% Ce exhibited smaller grain size and lower roughness, whereas 5% Ce-doped films showed grain growth and increased roughness. Pure ZnO films displayed high transparency (>90%), whereas Ce incorporation caused a red shift in the absorption edge and narrowing of the optical band gap due to defect-related states and lattice distortion. Photocatalytic experiments revealed that Ce doping improved charge carrier separation and increased the number of oxygen vacancies. Among all samples, the 2% Ce-doped ZnO film demonstrated the highest photocatalytic efficiency. These findings highlight the importance of controlled Ce doping in tuning the microstructure, optical properties, and photocatalytic performance of ZnO thin films, making them suitable for environmental remediation and optoelectronic applications.

## 1. Introduction

ZnO has a wide band gap of approximately 3.37 eV and is considered one of the most appealing and interesting semiconductor materials due to its excellent optical properties, chemical stability, low toxicity, and cost-effectiveness. All these traits and characteristics make it a strong candidate for various optoelectronic applications. It is commonly used in devices such as sensors, solar cells, transparent conductive oxides, and photocatalyst layers. But how well ZnO thin films perform really depends on how they are grown, what their surface looks like, what kind of dopant you use and how much of that dopant you add. It is strongly influenced by the growth processes, film morphology, dopant type, and dopant concentration. Therefore, substantial research has focused on doped ZnO structures and engineered layers that enable enhanced functional properties. Adding rare-earth ions such as cerium (Ce) into ZnO can change its structural, optical, and defect properties. Specifically, it boosts photon absorption and helps generate more electron–hole pairs, which is important for photocatalysis applications [1,2,3,4].

Recent studies have demonstrated that both the optical properties and photocatalytic activity of Ce-doped ZnO thin films prepared by various deposition methods can be improved. Adding cerium affects the band-gap energy and optical absorption behavior, enhancing light-harvesting capability for visible-light photocatalysis and UV shielding [5,6]. Studies on co-doped ZnO systems suggest that Ce can introduce defect states and alter both refractive and electronic properties, emphasizing its potential use in optoelectronic devices and energy-conversion applications [4].

For the present study, thermal evaporation was utilized as a straightforward physical vapor-deposition method. It gives us high-purity, uniform thin films with well-controlled thickness, advantageous for systematically investigating dopant effects. This technique has been widely used to study functional thin films, helping researchers to establish relationships between deposition conditions, microstructure, and optical properties in chalcogenide and polymer composite systems [7,8,9,10,11]. These studies highlight the significance of controlled thin-film growth and dopant incorporation in tailoring light–matter interactions and functional performance. However, research on Ce-doped ZnO remains limited, with most studies focusing on powdered materials or thin films prepared by sol–gel, spray pyrolysis, or co-doping methods [12,13,14,15,16,17]. One promising application of Ce-doped ZnO thin films is environmental purification, particularly the photocatalytic degradation of organic pollutants. Photocatalysts based on ZnO are widely used for the decomposition of hazardous organic compounds, including dyes, pesticides, and pharmaceutical residues, under UV or near-UV irradiation due to the generation of reactive oxygen species [18,19]. Cerium incorporation significantly improves photocatalytic activity by introducing defect states and oxygen vacancies, improving light absorption and suppressing electron–hole recombination [20,21]. Ce-doped ZnO thin films also show improved charge carrier separation and extended carrier lifetimes, enabling more efficient degradation of pollutants with lower energy consumption [22,23].

In this work, pure ZnO and 2% and 5% Ce-doped ZnO thin films were fabricated using thermal evaporation. The effects of cerium concentration on structural, morphological, optical, and photocatalytic properties were systematically investigated to provide insight into the design of efficient ZnO-based photocatalytic thin films. Although Ce-doped ZnO has been studied extensively, the novelty of present work is different in terms of its fabrication route and systematic characterization analysis. While most published reports rely on chemical synthesis methods, this study presents a rather classical physical deposition technique, vacuum thermal evaporation, along with exploring less frequent up to now based studies related to Ce-doped ZnO thin films. In addition, a comparative study between pure ZnO and Ce-doped ZnO (2% and 5%) thin films is reported, allowing for an easy assessment on how cerium incorporation affects structural, optical, and photocatalytic properties. These findings offer a perspective on the significance of defect states originating from Ce and their effect on charge carrier dynamics as well as photocatalytic activity. Overall, they give us a solid understanding of how the doping level influences both the deposition method and the functional properties of ZnO-based thin films.

## 2. Experimental

### Materials and Methods

Pure ZnO thin films and ZnO films doped with 2% and 5% cerium (Alfa Aesar, Karlsruhe, Germany) were deposited onto glass substrates using a thermal vacuum evaporation with a B30 Hochvakuumpumpe Dresden system (VEB Hochvakuum Dresden, Dresden, Germany). The Ce-doped ZnO samples were prepared with appropriate weight ratios to obtain doping concentrations of 2% and 5% (for Pure ZnO 0.100 g ZnO; for 2% Ce-doped ZnO: 0.002 g CeO_2_ and 0.098 g ZnO; for 5% Ce-doped ZnO: 0.005 g CeO_2_ and 0.095 g ZnO). Prior to deposition, the starting composition was previously treated mechano-chemically with 1100 rpm; what is more, the powder mixtures were milled for several hours in a centrifugal ball mill (Retsch GmbH, Haan, Germany) to obtain a homogeneous composition. The prepared powders were then placed in a tantalum boat, while carefully cleaned glass substrates were positioned inside the vacuum chamber. The distance from the evaporator to the substrate was set at 12 cm. During the deposition a pressure of 10^−4^ Pa was achieved and maintained, which allowed the evaporated material to condense onto the substrates. Short exposures were used. The primary objective was to obtain monomolecular thin films at low temperatures; however, oxide decomposition may occur during subsequent heat treatment. For this reason, after deposition the films were annealed at 400 °C for 90 min.

Structural analysis was carried out at room temperature using a Philips APD-15 X-ray diffractometer (Philips Analytical, Eindhoven, The Netherlands) within a 2θ range of 20–85°, employing Cu Kα radiation (λ = 1.54178 Å). The surface morphology of the films was investigated by scanning electron microscopy (SEM) using a Raith eLine system (Raith GmbH, Dortmund, Germany), while their chemical composition was determined via energy-dispersive spectroscopy (EDS) with a Zeiss Smart EDX detector (Carl Zeiss Microscopy GmbH, Jena, Germany). Surface topography and roughness were further examined using atomic force microscopy (AFM), performed with a DualScope 95 SPM system (DME Danish Micro Engineering A/S, Copenhagen, Denmark). Optical properties were evaluated at room temperature using a Jasco V670 UV–VIS spectrophotometer (Jasco International Co., Ltd., Tokyo, Japan). Film thickness was measured using a Plasmos SD-2100 ellipsometer (PLASMOS GmbH, Munich, Germany) and additionally verified through SEM analysis with Hitachi S-4000 and S-4100 microscopes (Hitachi High-Tech Corporation, Tokyo, Japan).

Photocatalytic degradation experiments were conducted in a 500 mL thermostatic reactor equipped with a magnetic stirrer. The photocatalyst was placed horizontally 1 cm below the solution surface and illuminated with a 300 W Hanau quartz lamp providing indirect sunlight at an intensity of 6500 lx (Quarzlampen Gesellschaft mit beschränkter Haftung Hanau, Hanau, Germany), measured with a Mastech MS6610 luxmeter (MASTECH Co., Ltd., Dongguan, Guangdong, China). The concentration of methyl orange (MO) was determined by measuring solution transmittance at the wavelength of minimum transmission (λmin = 464 nm) using the Jasco V670 UV–VIS spectrophotometer [20].

## 3. Results and Discussion

### 3.1. Structural Study

**Figure 1:**
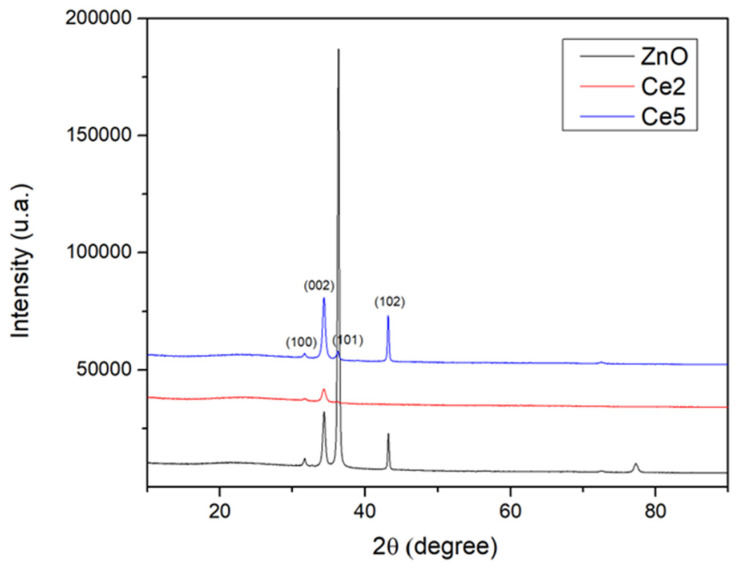
X-ray diffraction patterns of pure and 2% and 5% Ce-doped ZnO thin films.

Figure 1 shows the X-ray diffraction patterns of pure ZnO and Ce-doped ZnO thin films with Ce concentrations of 2% and 5%. All observed diffraction peaks correspond to the hexagonal wurtzite phase of ZnO, indicating that the wurtzite structure is preserved after Ce incorporation. No additional peaks related to Ce, CeO_2_, or other secondary phases were detected, suggesting that Ce ions are incorporated into the ZnO lattice without forming additional phases [23]. The strong diffraction peak associated with the (002) plane indicates a preferred c-axis orientation perpendicular to the substrate surface [24].

With increasing Ce content, the diffraction peaks decrease in intensity and broaden, indicating reduced crystallite size and increased lattice strain. This behavior arises from lattice distortion caused by substitution of Zn^2+^ ions by larger Ce^3+^/Ce^4+^ ions [25]. What is more, this limits the direct substitution into highly ordered zinc oxide lattice. Therefore, Ce incorporation is favored in less structurally constrained forms of matter such as thin films, nanoparticles or grain boundaries [26,27], where lattice distortions and defects can accommodate the size mismatch. At the highest doping level (5% Ce), the reduction in crystallinity suggests that Ce concentration is approaching its solubility limit in ZnO.

The ionic radius of Zn^2+^ (0.74 Å) is significantly smaller than those of Ce^4+^ (0.87 Å) and Ce^3+^ (1.01 Å). Incorporation of larger Ce ions into the ZnO lattice produces strain, consistent with peak broadening and slight peak shifts observed in the XRD patterns, confirming substitutional doping. The absence of CeO_2_-related peaks further supports the lack of secondary phase formation within the studied concentration range. The enhanced lattice strain observed at 5% Ce suggests that Ce^3+^ becomes the predominant oxidation state under these conditions [25].

### 3.2. Morphological Study

**Figure 2:**
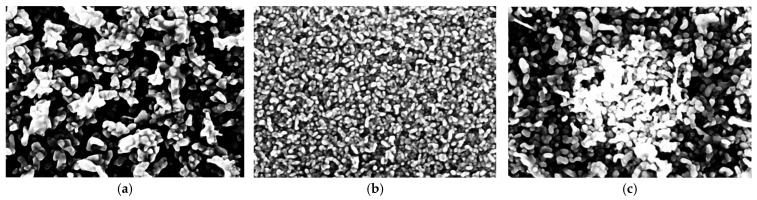
SEM images of (**a**) pure ZnO, (**b**) 2% Ce-ZnO and (**c**) 5% Ce-ZnO.

Figure 2 shows SEM images of pure ZnO, 2% Ce-doped ZnO, and 5% Ce-doped ZnO thin films. Looking first at the pure ZnO film, it has a granular morphology composed of nanoparticles with irregular shapes and a non-uniform grain-size distribution. The presence of loosely packed grains indicates limited nucleation density and unrestricted grain growth during evaporation. That in turn leads to a moderately rough surface [28,29]. Incorporation of 2% Ce results in a more compact and homogeneous morphology. The grains are finer and spread evenly across the surface. The reduced grain size and lower void density suggest that Ce ions enhance nucleation and suppress excessive grain growth. Ce ions substituting Zn^2+^ or occupying interstitial sites introduce lattice strain that modifies growth kinetics, thereby increasing nucleation density [25,30]. This behavior is consistent with previous reports demonstrating grain refinement in metal-doped ZnO thin films [31].

At 5% Ce, the morphology deteriorates. SEM images show agglomerated grains, clustered regions, and increased surface roughness. Bright contrast areas may indicate segregation of Ce-rich regions or secondary-phase formation due to the limited solubility of Ce in ZnO [32]. Excessive Ce incorporation disrupts crystal growth, causing grain coalescence and greater structural disorder. Similar effects at high dopant levels have been reported elsewhere [33,34].

Overall, SEM analysis confirms that Ce strongly influences ZnO microstructure. An optimal concentration of 2% improves grain refinement, film compactness, and surface uniformity, while 5% Ce leads to agglomeration and reduced morphological quality. These changes are expected to impact on the optical behavior of the films [35].

**Figure 3:**
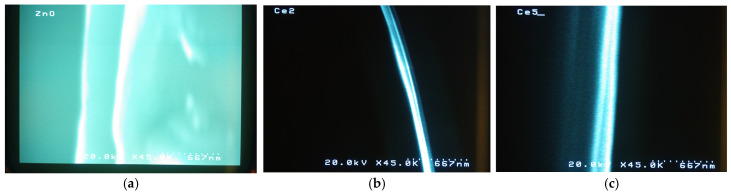
SEM images presenting the thickness of (**a**) pure ZnO (**b**) 2% Ce-ZnO and (**c**) 5% Ce-ZnO.

Figure 3 shows cross-sectional SEM images of pure ZnO, 2% Ce-doped ZnO, and 5% Ce-doped ZnO thin films. In all cases, you can see a uniform and continuous layer deposited on the substrate, suggesting good film coverage and adhesion. The pure ZnO film shows a relatively smooth profile with a well-defined thickness. As for the measured thicknesses: the pure ZnO film came out at 400 nm, 440 nm for 2% Ce-ZnO and 450 nm for 5% Ce-ZnO. Adding Ce does not change the uniformity much. However, slight variations in contrast and edge definition are observed, particularly for the 5% Ce-doped ZnO film. These changes can be attributed to the incorporation of Ce ions, which influences the growth of kinetics and microstructural evolution of films. These notable enhancements in roughness and contrast at higher Ce concentration relate with our findings of increased lattice strain and reduced crystallite size through the XRD analysis.

An ellipsometer was used in order to confirm the film thickness with another method. The results are presented in Table 1.

The ellipsometer and cross-sectional analysis results are quite similar, with small discrepancies between the two methods. These differences in morphology likely resulted from the film’s refractive index, surface morphology and Ce doping influence on the growth process. Overall, the film thickness increases with Ce doping, from 420 nm for pure ZnO to 480 nm for 5% Ce-ZnO, indicating the influence of Ce incorporation on the film’s growth characteristics.

**Figure 4:**
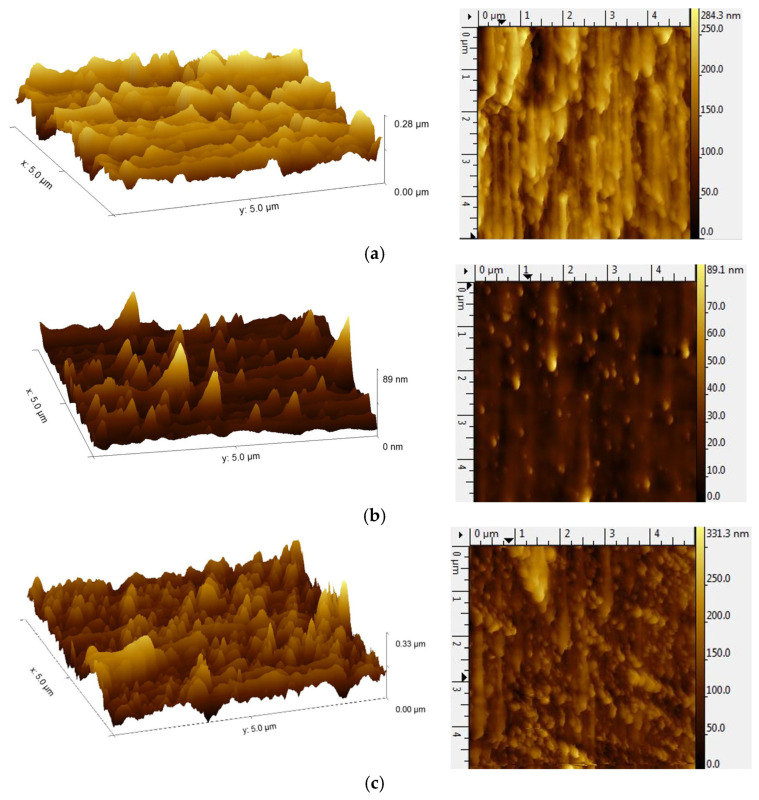
AFM 3D and 2D images of (**a**) pure ZnO thin films, (**b**) 2% Ce-ZnO and (**c**) 5% Ce-ZnO thin films.

AFM analysis reveals substantial differences in surface roughness, as shown in Figure 4. Pure ZnO exhibits an RMS roughness of 39.02 nm, showing an uneven grain growth.

The 2% Ce-doped ZnO film, on the other hand, has a much smoother surface with an RMS roughness of 7.52 nm that indicates that doping with Ce improves its nucleation and produces a more condensed grain structure. However, at a high doping concentration of 5%, the surface roughness increases to 35.57 nm again with larger grain features on it, which could result from dopant segregation. The results suggest that 2% Ce beneficial to enhance surface morphology, high uniformity and smoothness with fine microstructure, while 5% Ce doping leads to a rougher surface in accordance with the SEM images.

### 3.3. Optical Properties

**Figure 5:**
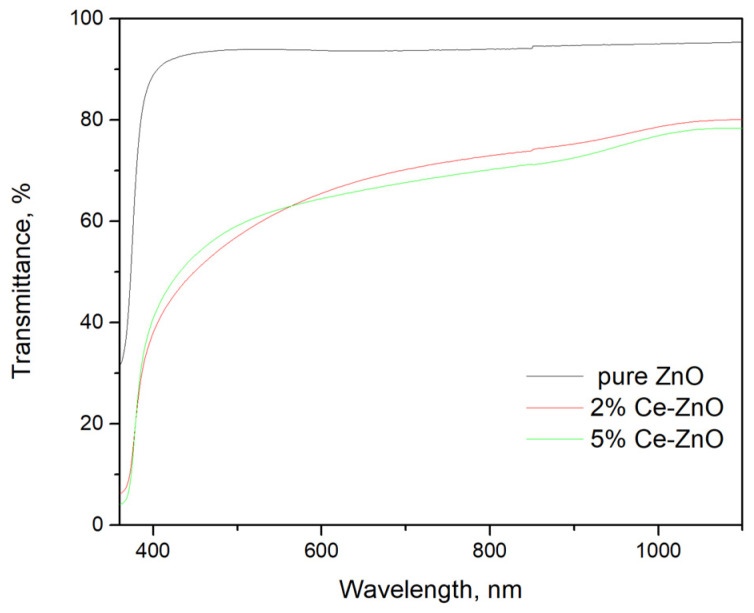
Optical transmittance spectra of pure and Ce-doped ZnO thin films.

Figure 5 presents the optical transmittance spectra of pure ZnO and Ce-doped ZnO thin films. The pure ZnO film exhibits high optical transparency in the visible and near-infrared regions, exceeding 90%, which is typical for ZnO films with low defect density and minimal light scattering [35,36].

In contrast, Ce-doped ZnO films show reduced transmittance, with values decreasing as Ce concentration increases. This behavior is attributed to the introduction of Ce-related defect states, increased surface roughness, and enhanced light scattering arising from lattice distortion and grain agglomeration, particularly at higher doping levels [37,38].

**Figure 6:**
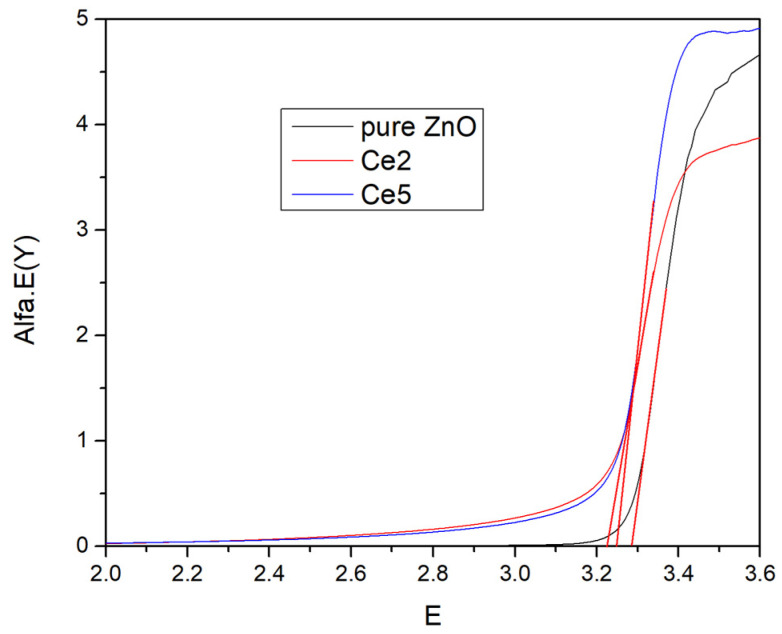
Band gap of pure and Ce-doped ZnO thin films.

Figure 6 shows the Tauc-plot-derived optical band gap energies. Pure ZnO displays a sharp absorption edge with a band gap near 3.3 eV. Ce doping results in a red shift in the edge and band gap narrowing for 2% and a slight increase for 5% Ce films. The incorporation of Ce-related impurity states and defect levels in the ZnO band structure can be related to such behavior. From the present data, it cannot be convincingly concluded what is the precise oxidation state of cerium (Ce^3+^/Ce^4+^). In the literature, it has been reported that Ce^3+^ ions are responsive to localized states near the conduction band and defect-induced band tailing can lead to a decrease in the band gap. However, Ce^4+^ ions can alter electronic interactions in distinct ways such as introducing stronger lattice distortion and possible charge compensation mechanisms that can lead to band gaps widening at their higher dopant concentrations [39,40,41,42,43]. Moderate doping, 2% Ce, provides a favorable balance between reduced band gap and acceptable transparency, making these films promising for optoelectronic and photocatalytic applications [44].

## 4. Application

Cerium doping plays a significant role in enhancing the photocatalytic performance of ZnO thin films by modifying their defect structure and charge-carrier dynamics. Incorporation of Ce into the ZnO lattice promotes the formation of oxygen vacancies, which act as active sites for photocatalytic reactions and facilitate the adsorption of reactant molecules. These vacancies also improve charge separation by suppressing the recombination of photogenerated electron–hole pairs under UV illumination. In addition, Ce^3+^ ions can function as electron traps, prolonging charge-carrier lifetimes and increasing the likelihood of surface redox reactions, ultimately enhancing photocatalytic efficiency [20,21,29].

Photocatalytic degradation proceeds through light-induced excitation of the photocatalyst, generating electron–hole pairs that subsequently form highly reactive radical species. These radicals attack and decompose organic pollutants in the solution [41]. To evaluate the photocatalytic degradation rate, ZnO and Ce-doped ZnO thin films with an active surface area of 1 cm x 1 cm were used. The surface area of the photocatalyst significantly influences degradation efficiency, as a larger exposed surface provides more active sites, enhancing the reaction rate.

Photocatalytic activity was assessed using aqueous solutions of methyl orange (MO) at a concentration of 0.05 mM. The photocatalytic measurements were performed in a 200 mL solution containing an initial methyl orange (MO) with time-resolved data collected at 10 min intervals under continuous illumination. The calculated degradation rates for pure ZnO and Ce-doped ZnO thin films after identical thermal treatment (90 min) are summarized in Table 2. The results indicate that Ce doping significantly influences the photocatalytic behavior of ZnO thin films, with dopant concentration affecting the balance between charge carrier generation, separation, and surface reaction kinetics.

## 5. Conclusions

In this study, pure ZnO and Ce-doped ZnO thin films (2% and 5% Ce) were successfully deposited on glass substrates via thermal vacuum evaporation, and their structural, morphological, optical, and photocatalytic properties were systematically investigated. X-ray diffraction confirmed that all films retained the hexagonal wurtzite structure of ZnO. Ce incorporation induced lattice strain and reduced crystallite size, with higher doping levels (5% Ce) approaching the solubility limit, resulting in partial deterioration of crystallinity. SEM and AFM analyses revealed that 2% Ce doping improved film compactness, grain refinement, and surface uniformity, whereas 5% Ce led to grain agglomeration and increased surface roughness due to dopant segregation and lattice distortion. Pure ZnO exhibited high visible transparency, while Ce doping reduced transmittance and caused band gap narrowing due to the formation of Ce-related defect states and lattice disorder. Ce doping enhanced charge carrier separation and promoted the formation of oxygen vacancies, significantly improving the photocatalytic degradation of methyl orange. Among the films studied, 2% Ce-ZnO exhibited the highest photocatalytic efficiency, demonstrating an optimal balance between structural quality, surface morphology, and optical absorption. These results demonstrate that controlled Ce doping effectively tunes the microstructural, optical, and photocatalytic properties of ZnO thin films, making them promising candidates for environmental remediation and optoelectronic applications. Future work would focus on exploring the integration of Ce-doped ZnO thin films into practical devices, such as photoreactors or optoelectronic systems, to further evaluate their real-world applicability.

## Figures and Tables

**Table 1:** Comparison of the film thicknesses measured with cross section and with ellipsometer.

Thin Film	Thickness Measured with Ellipsometer	Thickness Measured with Cross Section
Pure ZnO	420 nm	400 nm
2% Ce-ZnO	433 nm	440 nm
5% Ce-ZnO	480 nm	450 nm

**Table 2:** Photocatalytic degradation rates of methyl orange (MO) for pure ZnO and Ce-doped ZnO thin films after thermal treatment at 90 min.

Sample	Thermal Treatment, min	r (×10^−5^ mM dm^−3^ min^−1^)
Pure ZnO	90	1.36
2% Ce-ZnO	90	7.76
5% Ce-ZnO	90	2.01

## Data Availability

The original contributions presented in the study are included in the article. Further inquiries can be directed at the corresponding author.
